# Dinoflagellate Phylogeny as Inferred from Heat Shock Protein 90 and Ribosomal Gene Sequences

**DOI:** 10.1371/journal.pone.0013220

**Published:** 2010-10-08

**Authors:** Mona Hoppenrath, Brian S. Leander

**Affiliations:** 1 Department of Zoology, University of British Columbia, Canadian Institute for Advanced Research, Program in Integrated Microbial Biodiversity, Vancouver, Canada; 2 Forschungsinstitut Senckenberg, Deutsches Zentrum für Marine Biodiversitätsforschung, Wilhelmshaven, Germany; University of Poitiers, France

## Abstract

**Background:**

Interrelationships among dinoflagellates in molecular phylogenies are largely unresolved, especially in the deepest branches. Ribosomal DNA (rDNA) sequences provide phylogenetic signals only at the tips of the dinoflagellate tree. Two reasons for the poor resolution of deep dinoflagellate relationships using rDNA sequences are (1) most sites are relatively conserved and (2) there are different evolutionary rates among sites in different lineages. Therefore, alternative molecular markers are required to address the deeper phylogenetic relationships among dinoflagellates. Preliminary evidence indicates that the heat shock protein 90 gene (Hsp90) will provide an informative marker, mainly because this gene is relatively long and appears to have relatively uniform rates of evolution in different lineages.

**Methodology/Principal Findings:**

We more than doubled the previous dataset of Hsp90 sequences from dinoflagellates by generating additional sequences from 17 different species, representing seven different orders. In order to concatenate the Hsp90 data with rDNA sequences, we supplemented the Hsp90 sequences with three new SSU rDNA sequences and five new LSU rDNA sequences. The new Hsp90 sequences were generated, in part, from four additional heterotrophic dinoflagellates and the type species for six different genera. Molecular phylogenetic analyses resulted in a paraphyletic assemblage near the base of the dinoflagellate tree consisting of only athecate species. However, *Noctiluca* was never part of this assemblage and branched in a position that was nested within other lineages of dinokaryotes. The phylogenetic trees inferred from Hsp90 sequences were consistent with trees inferred from rDNA sequences in that the backbone of the dinoflagellate clade was largely unresolved.

**Conclusions/Significance:**

The sequence conservation in both Hsp90 and rDNA sequences and the poor resolution of the deepest nodes suggests that dinoflagellates reflect an explosive radiation in morphological diversity in their recent evolutionary past. Nonetheless, the more comprehensive analysis of Hsp90 sequences enabled us to infer phylogenetic interrelationships of dinoflagellates more rigorously. For instance, the phylogenetic position of *Noctiluca*, which possesses several unusual features, was incongruent with previous phylogenetic studies. Therefore, the generation of additional dinoflagellate Hsp90 sequences is expected to refine the stem group of athecate species observed here and contribute to future multi-gene analyses of dinoflagellate interrelationships.

## Introduction

Dinoflagellates comprise an extraordinary lineage of protists (unicellular eukaryotes) in regard to overall diversity in cell morphology and nutritional modes (e.g., phagotrophy, ‘klepto-phototrophy’, photoautotrophy, mixotrophy, and parasitism) [1]–[3]. Both heterotrophic and photoautotrophic members of the group are abundant and ecologically important components of marine planktonic communities. Dinoflagellates are morphologically distinct from other eukaryotes in the structure of their (dinokont) flagellar apparatus and (dinokaryotic) nucleus (i.e., permanently condensed chromosomes without typical eukaryotic histones and with an extranuclear spindle that passes through cytoplasmic channels) [1], [4], [5].

The monophyly of dinoflagellates and their phylogenetic relationships to other alveolate taxa, like ciliates and apicomplexans, have been demonstrated with several different molecular markers [1], [4], [6]–[11]. However, the interrelationships of the major subgroups of dinoflagellates are still unresolved using current molecular markers, mainly because of a lack of statistical support (i.e., phylogenetic signal) for the branching order near the phylogenetic backbone of the group [12]–[14]. The evolutionary relationships of dinoflagellates were initially inferred from a comparison of morphological characters [15], and these data are very important for evaluating weakly resolved branching patterns inferred from molecular markers [13], [16]. Accordingly, the poor phylogenetic resolution associated with the molecular markers employed so far prolongs our reliance on morphological characters when making inferences about dinoflagellate evolutionary history [12], [13], [16]. As such, inferences based on morphology have yet to be adequately tested with molecular markers that provide sufficient signal at the deepest levels in the dinoflagellate phylogenetic tree.

Ribosomal DNA (rDNA) sequences are most useful for resolving (“genus” level) relationships near the tips of the dinoflagellate tree [12], [17]–[21]. Deeper branches receive either no or poor statistical support in trees inferred from rDNA for several reasons: (1) a large number of highly conserved regions; (2) strong evolutionary rate heterogeneity among sites in variable regions; (3) high levels of compositional heterogeneity among some of the sequences; (4) high levels of homoplasy within variable regions; and (5) non-independently evolving sites in paired helix regions [12]–[14], [22]. Moreover, taxon sample biases and taxon identification are reoccurring problems – fewer than 150 species of about 2,500 known species have so far been sequenced, with a strong bias towards photosynthetic taxa [14]. Although some effort has been made to increase the representation of heterotrophic and uncultivated taxa in the datasets over the past five years [18], [19], [21], [23], the taxon bias remains.

Understanding the phylogeny of athecate (unarmored) dinoflagellates is particularly problematic because (1) their patterns of amphiesmal vesicles are more difficult to discern than in thecate (armored) dinoflagellates, (2) many of them are heterotrophic and uncultivated, (3) they are widely polyphyletic in molecular phylogenetic analyses, and (4) many of them have been misclassified [13], [14], [21], [24], [25]. Nonetheless, detailed re-evaluations of morphology combined with molecular phylogenetic studies of several athecate taxa over the past ten years has resulted in descriptions of new genera and improved re-descriptions of existing genera [12], [17], [20], [21], [23], [26]–[30].

The phylogenetic position of the (athecate) Noctilucales is especially controversial. These free-living dinoflagellates possess a dinokaryon only during part of their lifecycle and sometimes possess a highly distinctive trophont stage consisting of an inflated balloon-like cell with a feeding tentacle. Molecular phylogenetic analyses of rDNA sequences and heat shock protein gene (Hsp90) sequences plus the absence of a dinokaryon in the trophont stage suggested that *Noctiluca* was an early diverging lineage of dinoflagellates that retained several ancestral states for the group as a whole (e.g., a pre-dinokaryotic nucleus) [31]–[34]. However, the molecular phylogenetic position of *Noctiluca* is inconsistent in different analyses, and these cells possess several very novel morphological features, so some authors have questioned the interpretation that this lineage is basal among dinoflagellates [13], [16].

The major subgroups of dinoflagellates are largely recognized from patterns of either amphiesmal vesicles or thecal plates, called “tabulation patterns”. This morphology-based criterion has been used to identify several monophyletic groups of dinoflagellates, some of which have been corroborated with molecular phylogenetic data, such as the Suessiales, the Gonyaulacales, the Dinophysiales, the Prorocentrales, and the Gymnodiniales sensu stricto [12]–[14], [20], [21], [24], [29], [35], [36]. Several lineages previously classified within the “Gymnodiniales” have been removed from this subgroup upon closer examination with electron microscopy and molecular phylogenetic analyses [37]. The tabulation pattern found in the Suessiales (represented by *Polarella* and *Symbiodinium*) forms an intermediate between the tabulation patterns found in some athecate taxa (previously lumped within the Gymnodiniales) and several thecate subgroups, like the Peridiniales and the Gonyaulacales. Although taxon sampling is far from complete, molecular phylogenetic analyses indicate that the Peridiniales is paraphyletic and might form a stem group from which the Gonyaulacales, Dinophysiales and Prorocentrales evolved [13]. Moreover, the highly distinctive morphology of the Prorocentrales indicates that the group is monophyletic, but molecular phylogenetic data did not corroborate this inference [13], [14], [24], [38]–[40] until analyses of mitochondrial genes were performed [25], [41].

Along these lines, molecular phylogenetic analyses of mitochondrial gene sequences (*cob* + *cox1*) concatenated with SSU rDNA recover basal positions for *Amphidinium* (athecate) and *Heterocapsa* (thecate) [25]; some paleontological data also support this hypothesis [13]. Although the general morphology of *Amphidinium* and *Heterocapsa* does not immediately indicate a close relationship between them, both genera contain species that possess body scales [42]–[44]. Scales are unusual in dinoflagellates and are known only in these two genera plus *Lepidodinium* [45], [46]. Perhaps significantly, *Oxyrrhis*, which is a sister lineage to dinokaryotes (syn. “core” dinoflagellates), also possesses scales on the cell body and the flagella [11], [47]–[49]. The putative phylogenetic distribution of this character suggests that the most recent ancestor of *Oxyrrhis* and dinokaryotes also possessed body scales.

However, inferences about morphological character evolution in dinoflagellates depend on a robust molecular phylogenetic framework, especially at the deepest levels that relate the major subgroups (i.e., “orders”). Accomplishing this requires exploration of different molecular markers, which is the primary aim of this study. We have chosen to significantly expand the current heat-shock protein 90 (Hsp90) dataset for dinoflagellates by more than doubling the taxon sample in a manner that enhances broader representation of the major subgroups. Hsp90 is a highly conserved molecule that functions as a chaperone for protein folding and plays a key role in cellular signal transduction networks in all eukaryotes [50]. Stechmann and Cavalier-Smith [51] predicted that Hsp90 could become an important “universal” phylogenetic marker for eukaryotes because it is relatively long (1,800 bp) and evolves relatively uniformly in very different lineages. These authors advocated that Hsp90 should be sequenced from a broad selection of eukaryotic taxa and included within multi-gene phylogenetic analyses. The relatively homogenous branch lengths in trees inferred from Hsp90 sequences also helps reduce methodological artifacts associated with long-branch attraction.

Hsp90 datasets have been used previously for inferring dinoflagellate relationships [10], [33], [52], [53]. The first dinoflagellate Hsp90 sequences were used to examine the relationships between the three major alveolate subgroups, which resulted in a strongly supported framework [52]; a few subsequent studies have used Hsp90 sequences to address the internal phylogeny of dinoflagellates [10], [33], [53]. One of these studies used Hsp90 sequences to explore the evolution of plastid diversity within dinoflagellates, which reinforced that there were several independent plastid replacements as suggested earlier using comparative morphology and analyses of rDNA sequences [53], [54]. A concatenated analysis of SSU rDNA sequences with Hsp90 sequences demonstrated considerably higher statistical support values for almost all of the deep nodes when compared to trees inferred from SSU rDNA alone [53]. Most recently, Hsp90 gene sequences were used to evaluate the controversial phylogenetic position of *N. scintillan*s, and the authors of this study concluded that *N. scintillan*s diverges very early within dinoflagellates [33]. However, all of these studies were limited by the very few Hsp90 sequences available at the time.

In an attempt to better resolve some of the earliest branches in the dinoflagellate phylogenetic tree, we sequenced Hsp90 gene sequences from 17 different species of dinoflagellates, covering as many orders as possible; consequently, the Hsp90 gene data set for dinoflagellates was more than doubled. Moreover, in order to be able to concatenate the Hsp90 data with LSU and SSU rDNA sequences, we supplemented the new Hsp90 sequences with three new SSU rDNA sequences and five new LSU rDNA sequences. All of these data enabled us to address the broad phylogenetic interrelationships of dinoflagellates and will contribute to future analyses using larger multi-gene datasets.

## Results

New Hsp90 sequences were generated from dinoflagellates representing seven different orders, including the first sequences from the Phytodiniales and the Suessiales and from the genera *Akashiwo*, *Diplopsalis*, *Peridinium*, *Polarella*, *Protoperidinium*, *Scrippsiella*, *Spiniferodinium*, *Thecadinium* and *Togula*. Only three of the 12 previously known Hsp90 sequences from dinoflagellates were from heterotrophic species, namely *Crypthecodinium*, *Lessardia*, and *Noctiluca*; in this study, we generated four additional sequences from heterotrophic dinoflagellates, namely *Diplopsalis lenticula*, *Protoperidinium* sp., *P. steidingerae* and *P. crassipes*. An Hsp90 sequence from the phototrophic *Pyrocystis lunula* is available in GenBank, but the length of this sequence was too short to include it in our phylogenetic analyses. A sequence representing the type species of each genus is particularly important in dinoflagellates in order to maintain taxonomic stability in the phylogenetic trees. Accordingly, we generated new Hsp90 sequences from six type species: *Akashiwo sanguinea*, *Gymnodinium fuscum*, *Polarella glacialis*, *Spiniferodinium galeiformis*, *Thecadinium kofoidii* and *Togula britannica*. All 17 of the new Hsp90 sequences contained the diagnostic indel for dinoflagellates [10]. The new rDNA sequences generated in order to complete the combined phylogenetic analyses represent the first SSU rDNA sequences from *Amphidinium mootonorum* and *Spiniferodinium galeiformis* and the first LSU rDNA sequence from *Thecadinium kofoidii*.

**Figure 1:**
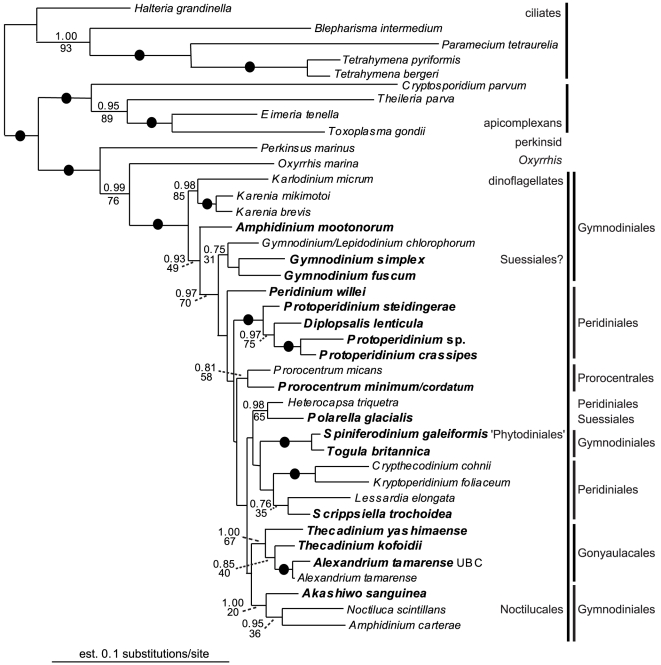
Bayesian tree inferred from 40 Hsp90 DNA sequences (3rd codon positions excluded; dataset 3), 984 unambiguously aligned sites and a GTR+I+G+8 model of nucleotide substitutions. Numbers above the branches denote ML bootstrap percentages, and numbers below the branches denote Bayesian posterior probabilities. Black circles denote bootstrap percentages and posterior probabilities of 100% and 1.00, respectively.

**Figure 2 pone-0013220-g002:**
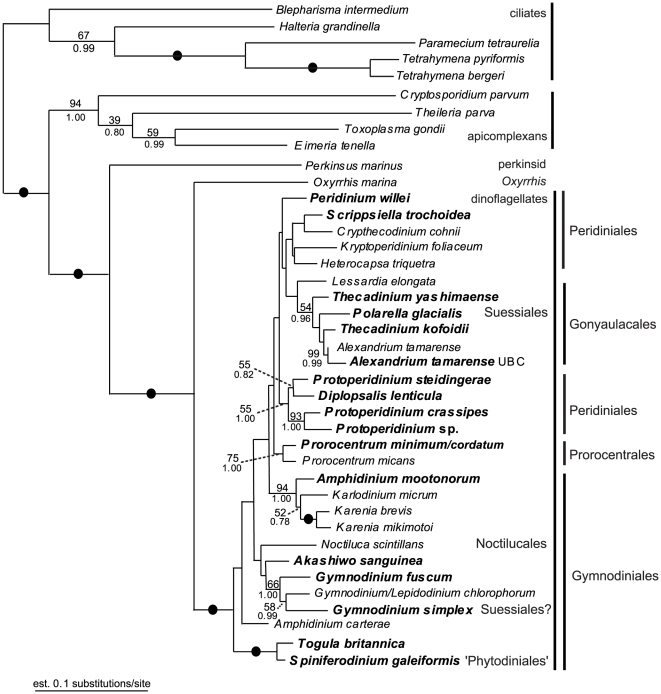
Maximum likelihood (ML) tree inferred from 40 Hsp90 amino acid sequences (dataset 4), 511 unambiguously aligned sites and a WAG model of substitutions. Numbers above the branches denote ML bootstrap percentages, and numbers below the branches denote Bayesian posterior probabilities. Black circles denote bootstrap percentages and posterior probabilities of 100% and 1.00, respectively.

**Figure 3 pone-0013220-g003:**
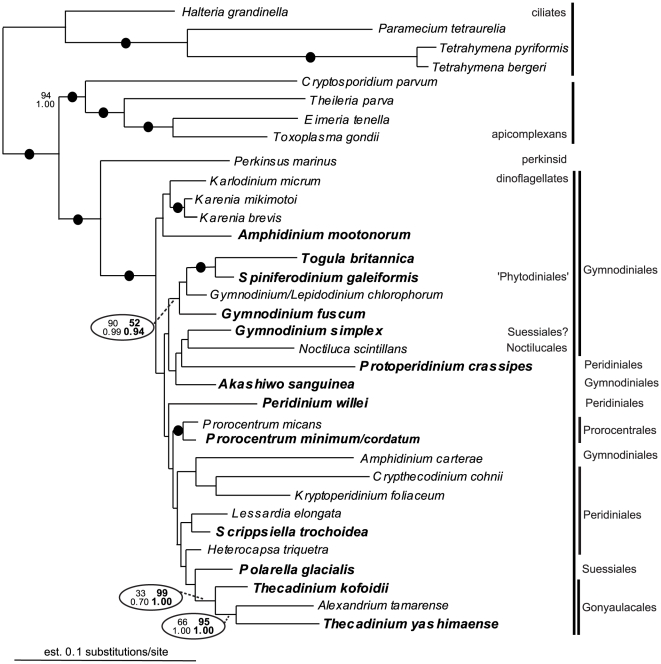
Bayesian tree inferred from 34 Hsp90 DNA sequences (3rd codon positions excluded) concatenated with SSU rDNA sequences (dataset 5), 2365 unambiguously aligned sites and a GTR+I+G+8 model of nucleotide substitutions. Numbers above the branches denote ML bootstrap percentages, and numbers below the branches denote Bayesian posterior probabilities. Black circles denote bootstrap percentages and posterior probabilities of 100% and 1.00, respectively. Numbers within the ovals compare the statistical support values from the analyses of dataset 5 (bold and to the right) and the analyses of the SSU rDNA sequences alone (dataset 1; to the left).

**Figure 4:**
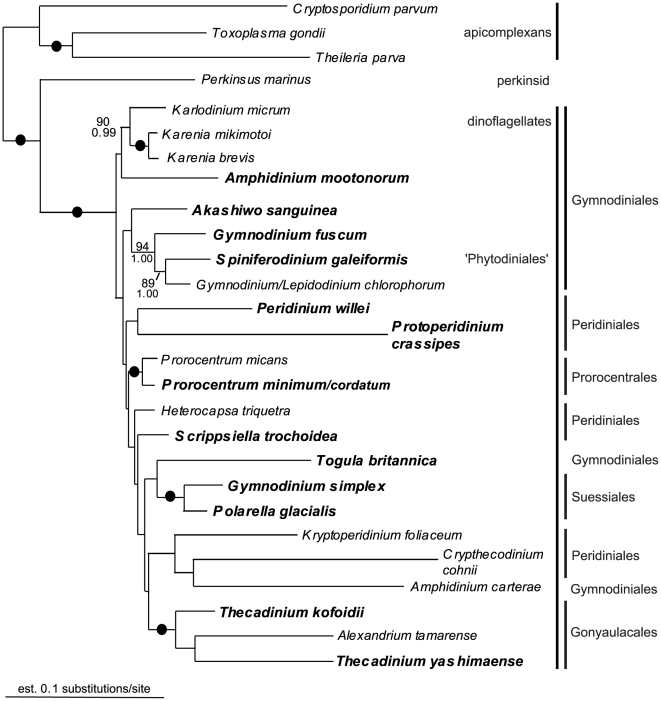
Bayesian tree inferred from 27 Hsp90 DNA sequences (3rd codon positions excluded) concatenated with SSU rDNA sequences and LSU rDNA sequences (dataset 6), 2847 unambiguously aligned sites and a GTR+I+G+8 model of nucleotide substitutions. Numbers above the branches denote ML bootstrap percentages, and numbers below the branches denote Bayesian posterior probabilities. Black circles denote bootstrap percentages and posterior probabilities of 100% and 1.00, respectively.

Six different alignments were constructed and analyzed: (1) SSU rDNA (35 taxa); (2) LSU rDNA (30 taxa); (3) Hsp90 DNA, 3rd codon positions excluded (40 taxa); (4) amino acid sequences inferred from the Hsp90 DNA sequences (40 taxa); (5) Hsp90 DNA, 3rd codon positions excluded, concatenated with SSU rDNA (34 taxa); and (6) Hsp90 DNA, 3rd codon positions excluded, concatenated with SSU rDNA and LSU rDNA (27 taxa). The resulting trees from datasets 3 to 6 are presented as Figures 1, 2, 3, 4, respectively. The statistical support values from the analyses of the SSU rDNA alone (dataset 1, Figure S1) were added to the corresponding nodes in Figure 3 (dataset 5). Analyses of the LSU rDNA sequences alone (dataset 2) resulted in a poorly resolved phylogeny (Figure S2).

The monophyly of dinoflagellates and dinozoans (i.e., the most recent ancestor of dinoflagellates and perkinsids and all of its descendants) received high support in all of the analyses (Figures 1–4). The statistical support values for basal nodes within dinokaryotes were low in all of the analyses, except for a few basal nodes in the tree inferred from dataset 3 (Hsp90 DNA, 3rd codon positions excluded) (Figure 1). The Gonyaulacales and the Prorocentrales received modest to strong support in all of the analyses, especially in trees inferred from datasets including rDNA sequences (Figures 3, 4). The *Protoperidinium/Diplopsalis* clade and the *Karenia* clade were strongly supported in trees inferred from Hsp90 sequences (Figures 1, 2). *Togula britannica* and *Spiniferodinium galeiformis* formed a strongly supported clade in trees inferred from Hsp90 sequences alone and in trees inferred from datasets including both Hsp90 and SSU rDNA (Figures 1, 2, 3). Unexpectedly, *Polarella glacialis* and *Gymnodinium simplex* did not cluster together in the trees inferred from datasets 3–5 (Figures 1, 2, 3) but did cluster strongly together in the tree inferred from dataset 6 (Figure 4).

Genera of athecate species branched as a paraphyletic assemblage near the base of the dinoflagellate tree in all of the analyses (Figures 1, 2, 3, 4). Moreover, in all of the trees inferred from DNA sequences, the *Karenia/Karlodinium* clade formed the earliest diverging lineage among the dinoflagellates included in the analyses (Figures 1, 3, 4); the tree inferred from amino acid sequences had an anomalous topology, whereby the *Togula/Spiniferodinium* clade formed the earliest diverging lineage (Figure 2). *Amphidinium carterae*, which is a representative of the *Amphidinium* sensu stricto, also branched near the base of the dinoflagellate tree in all of the analyses, albeit with weak statistical support (Figures 1–4). Nonetheless, neither *Noctiluca* nor *Heterocapsa* ever branched in a basal position relative to the other core dinoflagellates in the analyses (Figures 1, 2, 3, 4). Instead, *Noctiluca* branched in a position that was deeply nested within other lineages of dinokaryotes, especially within the trees inferred from Hsp90 DNA sequences (Figure 1). In order to gain additional insight into how well the Hsp90 data supported the phylogenetic position of *Noctiluca* relative to dinokaryotes, we performed AU tests for comparing the likelihoods of two alternative topologies differing in the relative position of this species: (1) *Noctiluca* positioned as shown in Figure 1, and (2) *Noctiluca* positioned as the nearest sister lineage to all dinokaryotes in the analysis (e.g., after *Oxyrrhis* and before the *Karlodinium*/*Karenia* clade, Figure 1). Topology 2 was strongly rejected by the AU test in the datasets that incorporated Hsp90 DNA sequences (*P* value for the AU test = 4×10^−6^) and topology 1 was supported (*P* value for the AU test  = 1.00).

## Discussion

### General phylogenetic patterns among athecate dinokaryotes

All trees inferred from the data generated in this study have nearly the same taxon composition in order to make the most direct comparison possible between the different phylogenetic markers employed. As outlined in the Results section, several topological differences were detected in trees inferred from Hsp90 sequences (including concatenations with rDNA, Figures 1, 2, 3, 4) and trees inferred from rDNA sequences alone (additional Figure 1 and published trees from previous studies). Some of these differences were also recognized in previous studies that explored Hsp90 as a phylogenetic marker for dinoflagellates [10], [53]. Shalchian-Tabrizi et al. [53] also noticed that although the branching order in trees inferred from SSU rDNA and Hsp90 sequences was generally congruent, the statistical support values for most of the deep nodes were considerably higher in the Hsp90 analyses. However, analyses of Hsp90 amino acid sequences produce topologies that are different from those derived from analyses using Hsp90 DNA sequences (excluding the third codon positions), which can be attributed to a more conserved and thus weaker level of phylogenetic signal in the amino acid dataset [55].

The authors of previous molecular phylogenetic studies of rDNA sequences concluded that the Gymnodiniales are polyphyletic and that loss of a theca occurred multiple times independently [13], [14], [16], [54]. Not surprisingly, this scenario is also reflected in our phylogenetic analyses of rDNA sequences and our analyses of Hsp90 DNA sequences concatenated with rDNA sequences (Figures 3, 4). Zhang et al. [25] suggested that either the *Amphidinium* sensu stricto (e.g., *A*. *carterae*) or *Heterocapsa* occupy the earliest diverging position among dinokaryotes. By contrast, Murray et al. [14] reported that (1) *Noctiluca* formed the earliest diverging branch in trees inferred from SSU rDNA, (2) *Akashiwo* formed the earliest diverging branch in trees inferred from LSU rDNA and (3) *Karlodinium* formed the earliest diverging branch in trees inferred from a combination of SSU and LSU rDNA. This last topology is consistent with our studies of Hsp90 sequences, whereby the *Karenia/Karlodinium* clade formed the earliest diverging branch among dinokaryotes in all of the analyses of DNA sequences (Figures 1, 3, 4). *Amphidinium* and *Akashiwo* never branched as the earliest diverging lineage, and *Heterocapsa* and *Noctiluca* were consistently nested more deeply within the tree of dinokaryotes (Figures 1, 2, 3, 4).

Our phylogenetic analyses of the Hsp90 amino acids (dataset 4, Figure 2) resulted in athecate genera (i.e., the Gymnodiniales) branching as a paraphyletic assemblage that encompassed the most recent ancestor of all dinokaryotes. Because our study contained 12 species from nine different genera of athecate dinoflagellates, this paraphyletic distribution of athecate dinoflagellates is particularly compelling; this phylogenetic pattern is also consistent with a previous study of Hsp90 sequences that contained representatives of four athecate genera [53]. Therefore, our new sequences and molecular phylogenetic analyses provide additional support for the hypothesis that the initial evolutionary radiation of dinoflagellates involved athecate dinoflagellates that subsequently gave rise to several thecate lineages, perhaps independently (e.g., the Prorocentrales, Gonyaulacales and Peridiniales). This hypothesis is also consistent with the phylogenetic results derived from dataset 3 (Figure 1), dataset 5 (Figure 3) and dataset 6 (Figure 4); these trees show *Karenia*, *Karlodinium*, *Gymnodinium*, and *Amphidinium mootonorum* branching as a paraphyletic assemblage that incorporates the most recent ancestor of all dinokaryotes. In some of the analyses, *Spiniferodinium* and *Akashiwo* were also part of this athecate assemblage (Figures 3, 4). However, the statistical support values for the nodes near the backbone of the trees inferred from all of the datasets were generally modest at best.

*Polarella glacialis* and *Gymnodinium simplex* were not members of the same clade in the trees resulting from datasets 3–5 (Figures 1, 2, 3), but these species formed a robust clade in the tree inferred from a concatenation of all three genes (Hsp90, SSU rDNA and LSU rDNA, dataset 6) (Figure 4). Other phylogenies suggest that *G. simplex* belongs into the Suessiales [13]; overall, a more confident placement of *G. simplex* requires, in part, a more detailed morphological investigation of this species.

### The molecular phylogenetic position of *Noctiluca*

There is significant debate about the phylogenetic position of *Noctiluca scintillans* among dinoflagellates, mainly because this lineage possesses an unusual collection of morphological features. The molecular phylogenetic analyses published so far (e.g., SSU rDNA, LSU rDNA, β-tubulin, and Hsp90) suggest that *N. scintillans* diverges very early within dinoflagellates, and most studies show this species branching as the nearest sister lineage to dinokaryotes. Some of the morphological features in this lineage (e.g., the absence of a nucleus with permanently condensed chromosomes in the trophont stage) have, accordingly, been interpreted as concordant evidence for a sister relationship between *Noctiluca* and dinokaryotes [31]–[33]. Moreover, the published Hsp90 sequence from *N. scintillans* was previously analyzed within the context of other dinoflagellate sequences and shown to be the first branch to diverge from the other taxa in the analyses [33]. However, these analyses were limited by the very small taxon sample available at the time. We were able to re-evaluate these analyses with a much larger sample of Hsp90 sequences from dinoflagellates and show that *N. scintillans* never occupied a basal position and was instead more deeply nested within dinokaryotes (Figures 1, 2, 3). AU tests provided additional support for this inference.

Although the previous molecular phylogenetic analyses suggesting a basal position for *N. scintillans* have been questioned by some authors [13], [16], several other authors have used this framework to (mis)interpret different aspects of the biology of *N. scintillans*. For instance, Fukuda and Endoh [33] stated that Liu and Hastings [56] discovered the most ancestral type of luciferase gene in *N. scintillans*. However, this was only one of two alternative interpretations posed by Liu and Hastings [56] and was based on the assumption that *N. scintillans* had already been demonstrated to be among the earliest diverging dinokaryotes. The alternative interpretation posed was that the condition in *N. scintillans* was a derived state in this lineage: “The ancestral system may have had two genes, which fused in *Noctiluca* …” [56].

Moreover, Fukuda and Endoh [32], [33] attempted to reconstruct the early evolution of dinokaryotes based on the properties of the gametes in *N. scintillans*; we think this approach is problematic for several reasons. First, a comparison of trees inferred from ribosomal DNA sequences to trees inferred from Hsp90 sequences with a sufficient taxon sample (i.e., this study) demonstrate that the phylogenetic position of this lineage within dinoflagellates has not been confidently established. Thus, at this time, the characters in *N. scintillans* cannot be interpreted to be ancestral for dinokaryotes as a whole. Second, these authors characterized their observations of *N. scintillans* as representing the complete life cycle of this species without accounting for previously reported discrepancies [32]. For instance, the authors describe the gametes as being isogamous and having two flagella that are visible with light microscopy [32]. However, TEM was required to demonstrate that the swarmer cells of *N. scintillans* had a distinctly heteromorphic flagellation, with one long flagellum and one very short flagellum oriented to the left side of the cell [57]. The short flagellum is not visible with light microscopy, which is why Zingmark [58] previously described the gametes as being uniflagellated.

Contradictory observations in the literature also led Schnepf and Drebes [59] to re-investigate sexual reproduction in *N. scintillans* and conclude that although a few microgametes with two flagella were present, generally the microgametes possess only a single longitudinal flagellum and do not undergo fusion. Schnepf and Drebes agreed with Uhlig [60], who reasoned that the appearance of gamete fusion and the presence of two long flagella is a consequence of incomplete cytokinesis. The possibility of an anisogamous (or nearly oogamous) sexual cycle was also suggested, but the author's explicitly stated that definitive evidence is unavailable [59]. Until the fusion of gametes and karyogamy is convincingly demonstrated, the mode of sexual reproduction in *N. scintillans* will remain speculative. The “isogamy hypothesis” and the transformation of the zygote into a mature trophont characterized by Fukuda and Endoh [32], [33] also need to be more convincingly described. Perhaps the best way to establish a more confident phylogenetic position and life cycle for the Noctilucales is to move beyond *N. scintillans* and characterize more species within the “order” at both the ultrastructural and molecular phylogenetic levels [61].

### Concluding Remarks

The resolution of interrelationships between the major lineages of dinoflagellates was modest at best when inferred from Hsp90 sequences alone or in concatenation with rDNA sequences. The high degree of sequence conservation and the consistently poor to modest resolution of the deepest nodes in trees inferred from Hsp90 and rDNA sequences supports the hypothesis that dinoflagellates underwent an explosive radiation in morphological diversity relatively recently in their evolutionary history. However, the lack of sufficient phylogenetic signal in the markers analyzed so far for dinoflagellates could be explained in other ways as well (e.g., mutational saturation over a large period of time). Nonetheless, the more comprehensive analysis of Hsp90 sequences presented here enabled us to re-address several phylogenetic interrelationships of dinoflagellates, such as the phylogenetic position of *N. scintillans*. Currently, there are no Hsp90 sequences available for the Dinophysiales, the Blastodiniales, and the Syndiniales, and the taxon sampling within the other “orders” is far from being an adequate representation for the overall biodiversity within these groups. In our opinion, the Hsp90 dataset for dinoflagellates should be expanded with the inclusion of *Dinophysis* species, *Pfiesteria*-like species, woloszynskioid species, additional noctilucoid species (e.g., *Spatulodinium* and *Kofoidinium*), and additional *Prorocentrum* species that represent the two separate clades inferred from rDNA phylogenies. Moreover, the incorporation of Hsp90 sequences from additional athecate taxa, like *Gyrodinium*, *Polykrikos*, *Takayama*, and *Apicoporus*, will help verify the main phylogenetic pattern we observed in this study, namely that athecate dinoflagellates form a paraphyletic assemblage that includes the most recent ancestor of all dinokaryotes. The generation of additional Hsp90 sequences will also contribute significantly to future multi-gene analyses of dinoflagellate interrelationships, and the present study is an essential step in that direction.

## Materials and Methods

### Strain collection and culture conditions

The strains used in this study were either (1) isolated from natural samples (e.g., the plankton or intertidal sand) and brought into culture or (2) acquired from culture collections and colleagues (see Table 1 and acknowledgments). The strains we isolated were collected from Helgoland, German Bight, North Sea, Germany [62], [63]; Boundary Bay, Vancouver, Canada; and Pachena Beach, Vancouver Island, Canada. Cultures were maintained at 17°C under low light conditions in f/2-medium [64].

**Table 1:** Information about the dinoflagellate species from which sequences were generated in this study.

Taxon	Source	DNA extraction	PCR primers
*Akashiwo sanguinea*	culture SCCAP K-1503, Helgoland isolate	CTAB	F4-R2 (Hsp90)
*Alexandrium tamarense*	culture NEPCC 592	CTAB	F4-R2 (Hsp90)
*Amphidinium mootonorum*	culture from MH †, Isolate from Pachena Beach, BC	Master Pure kit	F4-R2b (Hsp90), PF1-R4 (SSU), D1R-R2 (LSU)
*Diplopsalis lenticula*	culture from K. Gribble, M2reiso3	Master Pure kit	F4-R2 (Hsp90)
*Gymnodinium fuscum*	culture CCMP 1677	Master Pure kit	F4-R2b & F4-R2 (Hsp90)
*Gymnodinium simplex*	culture SAMS 1117/3	DNeasy kit, provided by R. Stern	F4-R2b (Hsp90)
*Peridinium willei*	culture NEPCC 815	CTAB	F4-R2b (Hsp90)
*Polarella glacialis*	culture CCMP 1383	Master Pure kit	F4-R2 (Hsp90)
*Prorocentrum minimum*	culture SCCAP K-1501, Helgoland isolate	Master Pure kit	F4-R2b (Hsp90)
*Protoperidinium crassipes*	culture from K. Gribble, MO65-PC-1split1	Master Pure kit	F4-R2b & F4-R2 (Hsp90)
*Protoperidinium steidingerae*	culture from K. Gribble, MV0802-2	Master Pure kit	F4-R2b & F4-R2 (Hsp90)
*Protoperidinium* sp.	isolate from Bamfield	Phenol/chloro.	F4-R3 & F6int-R2b (Hsp90)
*Scrippsiella trochoidea*	culture SCCAP K-1502, Helgoland isolate	CTAB	F4-R2 (Hsp90)
*Spiniferodinium galeiformis*	Boundary Bay isolate	CTAB	F4-R2b (Hsp90), PF1-R4 (SSU), D1R-R2 (LSU)
*Thecadinium kofoidii*	culture SCCAP K-1504, Helgoland isolate	CTAB	F4-R2 (Hsp90), PF1-R4 (SSU), D1R-R2 (LSU)
*Thecadinium yashimaense*	culture CCMP1890	Master Pure kit	F4-R2b (Hsp90), D1R-R2 (LSU)
*Togula britannica*	culture from MH †, Boundary Bay isolate	CTAB	F4-R2b & F4-R2 (Hsp90), D1R-R2 (LSU)

CCMP  =  Provasoli-Guillard National Centre for Culture of Marine Phytoplankton, Hsp90 =  heat shock protein 90 sequence, lsu  =  large subunit ribosomal DNA sequence, MH  =  Mona Hoppenrath, NEPCC  =  North East Pacific Culture Collection (now CCCM  =  Canadian Center for the Culture of Microorganisms), SAMS  =  Scottish Association for Marine Science (CCAP  =  Culture Collection of Algae and Protozoa), SCCAP  =  Scandinavian Culture Collection of Algae & Protozoa, ssu  =  small subunit ribosomal DNA sequence,

† =  dead/lost.

The cultures of heterotrophic dinoflagellates were grown at room temperature and normal daylight conditions on a plankton wheel at 1–2 rpm and fed with either the diatom *Ditylum brightwellii* (*Diplopsalis lenticula* and *Protoperidinium steidingerae*) or the dinoflagellate *Lingulodinium polyedrum* (*Protoperidinium crassipes*). Cultures were transferred every 5 to 7 days by pouring approximately one half of the culture into a new flask containing medium and prey cells. The food cultures were grown at 17°C under low light conditions in f/2-medium [64]. See Gribble and Anderson [18], [22] for details of the protocol used for strain isolation and culture establishment.

Cells from cultures received from culture collections were harvested immediately for DNA extraction.

### DNA extraction, PCR amplification, cloning, and sequencing

Cells were manually isolated or pelleted from the culture medium. Two different methods for DNA extraction were used over the years (Table 1). (1) Collected cells were suspended into 400 µl CTAB extraction buffer (1.12 g Tris, 8.18 g NaCl, 0.74 g EDTA, 2 g CTAB, 2 g Polyvinylpyrolidone, 0.2 ml 2-mercaptoethanol in 100 ml water) in 1.5 ml Eppendorf tubes. The tube was placed in a heat-block and incubated at 63°C for 20 min with several vigorous shakes in between. After separation with chloroform:isoamyl alcohol (24∶1), the aqueous phase was precipitated in 70% ethanol. Distilled water was added to the dry DNA pellets and the samples were stored in the freezer prior to PCR. (2) Genomic DNA was extracted from the cells using the MasterPure complete DNA and RNA purification Kit (EPICENTRE, Madison, WI, USA). The Hsp90, small subunit, and large subunit rDNA sequences were PCR amplified using puReTaq Ready-to-go PCR beads (GE Healthcare, Quebec, Canada), with an error rate of 1 per 20,000–40,000 bases, and primers were used as reported previously [21] and in Table 2. PCR products of the expected size were gel isolated and cloned into pCR2.1 vector using a TOPO TA cloning kit (Invitrogen Corporation, CA, USA). One clone was completely sequenced with ABI big-dye reaction mix using both vector primers, internal primers in both directions (for SSU) and some times specific internal primers designed for the taxon (for Hsp90).

**Table 2:** PCR primers used in this study.

Gene	Primer name	Primer sequence 5′-3′	Citation
Hsp90	F4	GGAGCCTGATHATHAAYACNTTYTA	this study
	F6int	AAYAARMMNAARCCNHTNTGGATG	this study
	R2	CGCCTTCATMATNCSYTCCATRTTNGC	[10]
	R2b	GCCTTCATDATNCKYTCCATRTT	this study
	R3	GATGACYTTNARDATYTTRTTYTGYTG	[10]
SSU	PF1	GCGCTACCTGGTTGATCCTGCC	[70] (modified)
	R4	GATCCTTCTGCAGGTTCACCTAC	[70] (modified)
LSU	D1R	ACCCGCTGAATTTAAGCATA	[71]
	R2	ATTCGGCAGGTGAGTTGTTAC	[19]

GenBank accession codes of the used new and already published sequences are shown in Table 3.

**Table 3:** Taxa and their accession numbers used for the different alignments and phylogenetic analyses.

Taxon	SSU rDNA	LSU rDNA	Hsp90 (Hsp+SSU)	Combined (Hsp+SSU)	Combined (Hsp+SSU+LSU)
**Ciliates, apicomplexans & ** ***Perkinsus*** ** (outgroups)**					
*Blepharisma* **	M97909	x	AY390395	x	x
*Cryptosporidium parvum*	AF093489	AE040725	AY423866	included	included
*Eimeria tenella*	EF210325	x	AAB97088	included	x
*Halteria grandinella*	AY00744	x	AY391253	included	x
*Paramecium tetraurelia*	EF502045	x	AAG00569	included	x
*Perkinsus marinus*	AF126013	AY876319	AY391259	included	included
*Tetrahymena bergeri*	AF364039	x	AY391257	included	x
*Tetrahymena pyriformis*	EF070254	x	AAG00567	included	x
*Theileria parva*	AF013418	AF218825	AAA30132	included	included
*Toxoplasma gondii*	M97703	L25635.1	AAQ24837	included	included
**Dinoflagellates & ** ***Oxyrrhis*** ** (ingroup)**					
*Oxyrrhis marina*	x	x	AAR27544	x	x
*Akashiwo sanguinea*	AF276818	AF260396	**GU295192**	included	included
*Alexandrium tamarense*	AB088333	AY438021	AM184118	included	included
*Alex. tamarense* UBC	x	x	**GU295210**	x	x
*Amphidinium carterae*	AF274251	AY455669	EU876701	included	included
*Amphidinium mootonorum*	**GU295202**	**GU295205**	**GU295199**	included	included
*Crypthecodinium cohnii*	M64245	FJ939575	AAM02974	included	included
*Diplopsalis lenticula*	x	EF152794	**GU295193**	x	x
*Gymnodinium chlorophorum*	AM184122	AF200669	AM184119	included	included
*Gymnodinium fuscum*	AF022194	AF200676	**GU295194**	included	included
*Gymnodinium simplex*	DQ388466	AF060901	**GU295211**	included	included
*Heterocapsa triquetra*	AF022198	AF260401	AAR27541	included	included
*Karenia brevis*	AF172714	AF200677	AM184117	included	included
*Karenia mikimotoi*	AF022195	AF200682	AM184120	included	included
*Karlodinium micrum*	AF172712	AF200675	AM184121	included	included
*Kryptoperidinium foliaceum*	AF274268	EF052684	AAV32830	included	included
*Lessardia elongata*	AF521100	x	AY391256	included	x
*Noctiluca scintillans*	AF022200	x	AB297471	included	x
*Peridinium willei*	AF274272	AF260384	**GU295195**	included	included
*Polarella glacialis*	AF099183	AY571373	**GU295196**	included	included
*Prorocentrum micans*	M14649	AF260377	AAR27546	included	included
*Prorocentrum minimum*	AY421791	AF260379	**GU295201**	included	included
*Protoperidinium crassipes*	AB261515	EF152846	**GU295197**	included	included
*Protoperidinium steidingerae*	x	DQ444231	**GU295198**	x	x
*Protoperidinium* sp.	x	x	**GU295212**	x	x
*Scrippsiella trochoidea*	AF274277	AF260393	**GU295213**	included	included
*Spiniferodinium galeiformis*	**GU295203**	**GU295206**	**GU295214**	included	included
*Thecadinium kofoidii*	**GU295204**	**GU295207**	**GU295215**	included	included
*Thecadinium yashimaense*	AY238477	**GU295209**	**GU295200**	included	included
*Togula britannica* UBC	x	**GU295208**	**GU295216**	included	included
*Togula britannica*	AY443010	AY455679	X	included	included

**B. intermedium for Hsp90; *B. americanum* for SSU rDNA.

Accession numbers indicated in bold denotes sequences generated in this study.

### Molecular phylogenetic analyses

Six different alignments were constructed for phylogenetic analysis (Table 3): (1) SSU rDNA (35 taxa and 1,381 unambiguously aligned characters); (2) LSU rDNA (30 taxa and 482 unambiguously aligned characters); (3) Hsp90 DNA, first two codon positions (40 taxa and 984 unambiguously aligned characters); (4) amino acid sequences inferred from the Hsp90 DNA sequences (40 taxa and 511 unambiguously aligned characters); (5) Hsp90 DNA, first two codon positions, concatenated with SSU rDNA (34 taxa and 2,365 unambiguously aligned characters); and (6) Hsp90 DNA, first two codon positions, concatenated with SSU rDNA and LSU rDNA (27 taxa and 2847 unambiguously aligned characters). Unambiguously aligned sequences were confirmed by eye, and all gaps were excluded from the alignments prior to phylogenetic analyses.

Phylogenetic relationships were inferred from all six alignments using maximum likelihood (ML) and Bayesian inference (BI) methods with the programs RAxML v7.04 [65] and MrBayes v3.12 [66], [67], respectively. ML and BI analyses of the nucleotide alignments (i.e., alignments 1–3 and 5–6) were built under a GTR+I+G+8 model as suggested by the criteria implemented in ModelTest v0.1.1 [68]. Two alternative topologies differing in the relative position of *Noctiluca*, in the analyses of the Hsp90 DNA, were generated with TreeView. Approximately unbiased (AU) tests were performed with CONSEL [69] using the likelihoods calculated with RAxML v7.04 with the same models and parameters indicated above. ML and BI analyses of the amino acid alignment (i.e., alignment 4) was analyzed under a WAG model of substitution considering corrections for site-to-site rate variation (gamma) with eight categories of rate variation and proportion of invariable sites. In order to assess topological support, 500 bootstrap replicates were performed with RAxML on each alignment with the parameters described above.

Bayesian analyses consisted of two independent Markov Chain Monte Carlo (MCMC) runs of 2,000,000 generations were calculated with trees sampled every 50 generations and with a prior burn-in of 100,000 generations (i.e. the first 2,000 sampled trees were discarded). The convergence diagnostic for all six alignments was within 1.0 (±0.005). A majority rule consensus tree was constructed from 38,001 post-burn-in trees. Posterior probabilities correspond to the frequency at which a given node was found in the post-burn-in trees.

### Introns in the dinoflagellate Hsp90 sequences

Introns were present in only 3 of 17 hsp90 genes sequenced from genomic DNA. The hsp90 gene of *Peridinium willei* contained one canonical intron near the 5′ end of the template sequence between residues 467 and 563 (97 bases). The hsp90 gene of *Polarella glacialis* contained one non-canonical intron near the 5′ end of the template sequence between residues 112 and 245 (134 bases). The hsp90 gene of *Thecadiniium yashimaense* contained one canonical intron near the 5′ end of the template sequence between residues 355 and 643 (289 bases).

## Supporting Information

Figure S1 Maximum likelihood (ML) tree inferred from 35 SSU rDNA sequences (dataset 1), 1,381 unambiguously aligned sites and a GTR+I+G+8 model of nucleotide substitutions. Numbers above the branches denote ML bootstrap percentages, and numbers below the branches denote Bayesian posterior probabilities. Black circles denote bootstrap percentages and posterior probabilities of 100% and 1.00, respectively.
(.16 MB EPS)

Figure S2 Maximum likelihood (ML) tree inferred from 30 LSU rDNA sequences (dataset 2), 482 unambiguously aligned sites and a GTR+I+G+8 model of nucleotide substitutions. Numbers above the branches denote ML bootstrap percentages, and numbers below the branches denote Bayesian posterior probabilities. Black circles denote bootstrap percentages and posterior probabilities of 100% and 1.00, respectively.
(1.38 MB EPS)
